# Intramuscular Reactivity of the Modified Graphene Oxides and Their Bio-Reactivity in Aging Muscle

**DOI:** 10.3390/jfb16040115

**Published:** 2025-03-25

**Authors:** Xiaoting Jian, Jiayin Wang, Jijie Hu, Yangyang Li, Qisen Wang, Han Wang, Jingwen Huang, Yu Ke, Hua Liao

**Affiliations:** 1 Guangdong Provincial Key Laboratory of Construction and Detection in Tissue Engineering, Department of Anatomy, School of Basic Medical Sciences, Southern Medical University, Guangzhou 510515, China; jianxiaotingii@163.com (X.J.); liyyang0814@163.com (Y.L.); qsenaa@163.com (Q.W.); 17369320174@163.com (H.W.); hjw0112@163.com (J.H.); 2 Key Laboratory of Biomaterials of Guangdong Higher Education Institutes, Department of Biomedical Engineering, College of Life Science and Technology, Jinan University, Guangzhou 510632, China; karina1002@163.com; 3 Department of Orthopaedics and Traumatology, Nanfang Hospital, Southern Medical University, Guangzhou 510515, China; jjh@smu.edu.cn

**Keywords:** GO, muscle, PEG, PHBV, block copolymer micelle, inflammation

## Abstract

To enhance the biocompatibility and drug delivery efficiency of graphene oxide (GO), poly(ethylene glycol) (PEG), poly(3-hydroxybutyrate-co-3-hydroxyvalerate) (PHBV), or its triblock copolymer PEG-PHBV-PEG (PPP) were used to chemically modify GO. However, it is still unknown whether non-toxic polymer-modified GO mediates muscle toxicity or triggers intramuscular inflammation. This study aims to investigate the biological reactivity and inflammation/immune response induced by PEG, PHBV, or PPP modified GO when injected into the tibialis anterior (TA) muscle of mice prior to drug loading. The results showed that after muscle exposure, the coating of biocompatible polymers on GO is more likely to provoke muscle necrosis. Muscle regeneration was found to occur earlier and more effectively in muscle treated with hydrophilic PEG-GO and PPP-GO compared to muscle treated with hydrophobic PHBV-GO. When observing the transient muscle macrophage invasion of three modified GOs, PHBV-GO caused severe muscle necrosis in the early stage, induced a delayed peak of macrophage aggregation, and caused severe inflammatory progression. All three kinds of modified GO induced T cell aggregation to varying degrees, but PEG-GO induced early mass muscle recruitment of CD4^+^ T cells and was more sensitive to cytotoxic T cells. Based on the higher biocompatibility of PPP-GO in muscles, PPP-GO was implanted into the muscles of old or adult mice. Compared to adult mice, aged mice are more vulnerable to the stress from PPP-GO, as demonstrated by a delayed inflammatory response and muscle regeneration.

## 1. Introduction

Abundant oxygen-containing groups on the surface, high specific surface area, and excellent biocompatibility make graphene oxide (GO) a popular graphite derivative in biomedicine. These characteristics have given GO a prominent focus in the study of biomaterials for drug delivery systems [1,2,3,4,5]. Small molecule drugs, antibodies, DNA and proteins have been carried by GO. Researchers have shown that graphene nanomaterials can deliver drugs up to 200% more efficiently than other nanoparticle drug delivery systems [6,7]. Various non-toxic polymers have been utilized to modify GO in order to improve its properties, including biocompatibility and drug delivery efficiency. An instance of this is when GO is modified with biocompatible polyethylene glycol (PEG) through π–π interactions or chemical bonding in order to enhance the capacity for loading water-soluble medications [8]. Poly-3-hydroxybutyrate-3-hydroxypalladic acid (PHBV) was combined with GO to study its electrical conductivity, mechanical strength, and melt crystallization properties [9,10]. Biocompatibility can be improved by functionalizing GO with an amphiphilic block copolymer. In spite of this, the long chains of the copolymer can compromise the blade effect of GO, resulting in a reduction in its effectiveness as a drug delivery system. In our recent study, we addressed the issue by chemically modifying GO with PEG (PEG-GO), PHBV (PHBV-GO), or their triblock copolymer PEG-PHBV-PEG (PPP-GO). We assess and contrast their dispersibility, thermal characteristics, drug encapsulation and release patterns, cytotoxicity, and hemolytic activities. Our study suggests that GO modified with block copolymers demonstrates similar dispersibility and stability to GO modified with PEG. Furthermore, block copolymer modified GO shows a superior loading capacity for hydrophobic drugs in comparison to GO modified with PHBV. As a result of these findings, block copolymers are superior to homopolymers in terms of properties [11].

Research has examined the effects of GO nanomaterials on a wide range of systems, including respiratory, digestive, urinary, central nervous, reproductive, and developmental systems [12]. In mice treated with GO intratracheally, fibrosis developed in lung tissue, mitochondrial respiration increased, and reactive oxygen species were produced. This in turn activated inflammatory and apoptotic pathways in airway epithelial cells [13]. After being administered intravenously, GO has the potential to accumulate within the lungs, causing the formation of granulomas as well as pulmonary edema [14]. After intraperitoneal injection, GO polyethylene glycol functionalized derivatives accumulate mainly in the liver and spleen [15]. The interaction of free radicals and biomolecules in human plasma resulted in the formation of biocrowns on biodegraded GO nanosheets. The biotransformation of GO resulted in decreased production of reactive oxygen species and ultrastructural damage [16]. According to another study, GO adhered to and enveloped the chorion of zebrafish embryos, causing hypoxia and delayed hatching [17]. Microscopic analysis of tissue sections showed degeneration and necrosis in specific neuronal cells of the cerebral and cerebellar cortex in animals exposed to a high dose of GO [18]. Comprising nearly 40% of the body’s total mass, skeletal muscle serves as a significant source of potential immunogenic sites. Effective in triggering immune responses, intramuscular implantation has shown particular efficacy, such as in the administration of vaccines [19,20]. Among the various drug delivery routes, injection into muscle tissue remains a preferred method. It remains mostly unknown whether the nontoxic polymers modified GO mediates muscle toxicity or triggers intramuscular inflammation.

This study aims to examine the bio-reactivity and inflammation/immune responses triggered by PEG, PHBV, or PPP modified GO upon injection into the tibialis anterior muscle (TA) in mice prior to drug loading. In light of the growing elderly population, we also explored the bio-reactive properties of PPP-GO carriers in aged animals, offering insights for optimizing GO-dependent drug administration strategy for the elderly population.

## 2. Materials and Methods

### 2.1. Ethical Approval

Southern Medical University’s Laboratory Animal Center (Guangzhou, China) provided the Kunming mice (KM mice). KM mice were allowed to eat and drink freely under a constant temperature (23 °C) and humidity (50%) environment with a light-dark cycle of 12:12 h. All experimental methods and procedures were approved by the Animal Experimentation Ethics Committee of Southern Medical University. (Approval No. L2016068).

### 2.2. Synthesis of GO, PEG-GO, PHBV-GO and PPP-GO

Tianjin Chemical Reagents (Tianjin, China) provided the graphite powder, potassium permanganate (KMnO_4_), and phosphorus pentoxide (P_2_O_5_). Guangzhou Chemical Reagents (Guangzhou, China) supplied the sulfuric acid (H_2_SO_4_), hydrochloric acid (HCl), trichloromethane (CHCl_3_), N, N-dimethylformamide (DMF), n-hexane, petroleum ether, trifluoroacetic anhydride, methylene chloride, hydrogen peroxide (H_2_O_2_), and glacial acetic acid. J&K Chemical (Beijing, China) provided the poly (ethylene glycol) (PEG, *M_w_* = 2000 g/mol), diglyme (99.8%), ethylene glycol, anhydrous 1,2-dichloroethane (99.8%), dibutyltin (II) dilaurate (97.5%), and isophorone diisocyanate (IPDI, 98%). All chemicals were used as supplied without additional purification. Sigma-Aldrich (St. Louis, MO, USA) supplied the poly(3-hydroxybutyrate-co-3-hydroxyvalerate) (PHBV, *M_n_* = 1.85 × 10^5^) with 8 mol % HV content. Before use, the PHBV was dissolved in CHCl_3_, filtered, and precipitated in n-hexane. On the basis of our previous research, telechelic hydroxylated PHBV (PHBV-DIOL, *M_w_* = 4700 g/mol) and its triblock copolymer PPP were prepared [21,22]. GO was prepared using graphite powder as the raw material, and an improved Hummer method [23,24]. In short, graphite (1 g), P_2_O_5_ (1 g), and H_2_SO_4_ (23 mL) were combined and stirred in an ice bath for 1 h. Subsequently, KMnO_4_ (3 g) was introduced into the mixture, which was then magnetically stirred at 0 °C for 2 h and then at 35 °C for an additional 2 h. Following the addition of 100 mL of distilled water, agitate at a temperature of 80 °C for 30 min, lower the temperature to room level, and subject to treatment using the resulting H_2_O_2_. Proceed to cleanse multiple times with HCl and distilled water, utilize centrifugal force at a rate of 10,000 rpm for a duration of 10 min, and freeze-dry the substance to produce GO. A suspension of 200 mg of GO in 50 mL of anhydrous DMF was subjected to ultrasonic treatment for 1.5 h under a nitrogen atmosphere. To this mixture, 0.4 g of IPDI and 0.11 g of dibutyltin dilaurate (acting as a catalyst) were added, and the resulting suspension was stirred magnetically at 50 °C for 24 h. The IPDI-modified GO produced was then washed three times with DMF, centrifuged at 8000 rpm for 10 min, and freeze-dried. Subsequently, 0.1 g of the IPDI-modified GO was dispersed in 20 mL of DMF and ultrasonically treated for 30 min. Solutions of 10 mL of either PEG, PHBV-DIOL, or PEG-PHBV-PEG at a concentration of 0.01 g/mL in DMF were individually mixed with the IPDI-modified GO suspension under a nitrogen atmosphere, followed by magnetic stirring at 80 °C. After adding five to six drops of dibutyltin dilaurate to the suspension, the mixture was continuously stirred for 24 h, allowed to cool to room temperature, washed with DMF, and then freeze-dried. Samples were synthesized.

### 2.3. GO Nanoparticles Characterization

The chemical structure was analyzed by a EQUINOX 55 Fourier transform infrared spectrometer (FTIR, Bruker, Bremen, Germany) via the KBr pellet method. The spectra comprised 64 scans at a resolution of 1 cm^−1^ in 4000~400 cm^−1^. Crystalline structure was determined in a continuous mode by using a D8 ADVANCE X-ray diffraction meter (XRD, Bruker, Bremen, Germany) using Cu Kα radiation (λ = 0.15406 nm) with 2θ from 5° to 70° and pipe voltage of 40 kV.

Raman spectroscopy was used at room temperature using a Horiba LabRAM INV Raman microscope (HORIBA Jobin Yvon, Paris, France), which was outfitted with a 532 nm Nd:YAG laser, to examine the carbon makeup of the sample sheets.

The Scanning Electron Microscope (SEM) Flex 1000 (Hitachi, Chiyoda, Japan) was employed to analyze surface morphologies using an acceleration voltage of 20 kV. Before examination, the samples were coated with gold using sputter-coating. Transmission Electron Microscopy (TEM) images were captured by FEI Tecnai G^2^ F20 microscope (FEI, Hillsboro, OR, USA) at an acceleration voltage of 200 kV. To suspend the nanocomposites, 100% ethyl alcohol was employed in conjunction with ultrasound treatment for 1 h. For TEM sample preparation, the nanoparticles suspension was dropped onto a copper grid and subsequently dried at 70 °C for 30 min before observation.

The dispersibility experiment was conducted in the following manner: 10 mg of the nanocomposites were dispersed in 10 mL of deionized water, PBS, or DMEM using ultrasonic treatment for a duration of 180 min. Photographs were then taken to compare the stability of the nanocomposite suspensions at different time intervals.

### 2.4. GO Nanoparticles Muscle Implantation and Sample Collection

The study involved healthy young (6 to 8 weeks, 60 mice) or old (24 months, 20 mice) KM male mice. The mice received an intraperitoneal injection of 50 mg/kg 1% pentobarbital sodium as anesthesia. Subsequently, under aseptic conditions, the mice were administered 50 μL suspensions of GO, PEG-, PHBV-, and PPP-modified GO (5 mg/mL) into the TA muscle. Normal healthy mice were utilized as a control in this experiment. Euthanasia was performed at 4, 7, 10, 15, and 30 days after injection, and the TA muscle was collected.

### 2.5. Histological and Immunofluorescence Staining

The transection of TA muscle tissue (8 μm) was stained with hematoxylin and eosin (H&E) and immunofluorescence. Sections stained by immunofluorescence were first fixed with ice acetone (Guangzhou Chemical Reagents, Guangzhou, China) and then incubated with a solution containing 2% BSA (ACMEC, Shanghai, China). Following this, the sections were exposed to primary antibodies for overnight incubation at 4 °C. The antibodies used in this study included F4/80 monoclonal antibody (BM8) (Invitrogen, Carlsbad, CA, USA), CD11b monoclonal antibody (M1/70) (eBioscience, San Diego, CA, USA), MyoD Antibody—BSA Free (Novus Biologicals, Centennial, CO, USA), Desmin Monoclonal antibody (CST, Boston, MA, USA), Myogenin Monoclonal Antibody (F5D) (Invitrogen, Carlsbad, CA, USA), dystrophin polyclonal antibody (Proteintech Group, Inc., Chicago, IL, USA), and rabbit anti-myosin-3 polyclonal antibody (Bioss, Beijing, China).

In this research, we utilized secondary antibodies from Beyotime, China, namely, 488-goat anti-rabbit IgG (H+L), Cy3-goat anti-rat IgG (H+L), and 555-donkey anti-rabbit IgG (H+L). To label the nuclear materials, 4′,6-diamino-2-phenylindole (DAPI) (Abcam, Eugene, OR, USA) was employed. Staining images were examined and collected with a fluorescence microscope (Olympus BX51, Olympus Corporation, Tokyo, Japan).

### 2.6. Flow Cytometric Analysis

TA muscle was taken from mice and large nanoparticles were removed with tweezers, then the muscle was chopped up. The resulting muscle tissue samples were treated in 0.2% collagenase type II (Sigma-Aldrich, St. Louis, MO, USA) at 37 °C for 45 min. Small nanoparticles and large tissue blocks were removed by filter. The filtered cells were collected and homogenized in cold PBS (Solarbio, Beijing, China) and centrifuged (4 °C, 250 g, 5 min). Following the blockade of Fc receptors, the isolated cells were suspended in the primary antibody and incubated at room temperature for 20 to 30 min away from light. Multi-dimensional HD flow cytometry analyzer: LSRFortessa X-20 (BD, Franklin Lakes, NJ, USA), data analysis using FlowJo v10 software (BD, Franklin Lakes, NJ, USA). The antibodies utilized in this study were procured from eBioscience (San Diego, CA, USA) and included CD8α antibody (PE), CD4 antibody (Super Bright™ 436) and CD3ɛ antibody (APC). Additionally, anti-mouse F4/80 (PE), anti-mouse CD163 (APC), anti-mouse CD86 (PE-Cy5), and anti-mouse CD11b (FITC) antibodies were also employed.

### 2.7. In Vitro Test

C2C12 cells originating from ATCC in USA were grown in DMEM/F12 medium (HyClone, Logan, UT, USA) and were supplemented with 10% fetal calf serum (Gibco, San Diego, CA, USA), 100 units/mL of penicillin, and 100 μg/mL of streptomycin sulfate (Solarbio, Beijing, China). The cells were incubated at 37 °C in an incubator (Heraeus, Hanau, Germany) humidified with 5% CO_2_. C2C12 cells were treated with a DMEM medium containing 2% horse serum (Gibco, San Diego, CA, USA) for 72 h, differentiated into myotubes, and then co-cultured with different materials (concentration of 200 ug/mL) for 24 h, and the myotube morphology was observed under microscope.

### 2.8. Statistical Analysis

The data were presented as mean ± standard deviation (SD). One and two-way ANOVA were used for statistical analysis (GraphPad Prism 8.0.1(244)). The *p* value < 0.05 was considered statistically significant.

### 2.9. Single Sample Gene Set Enrichment Analysis (ssGSEA)

The source data is GSE217037. We downloaded and collated the data GSE217037 from the GEO database (https://www.ncbi.nlm.nih.gov/geo/, accessed on 1 November 2024). The count data was converted into TPM format, the gene set of Miao et al. [25] GSVA (v.1.50.5) R package was used for ssGSEA analysis, and then the ggplot2 (v.3.5.0) R package was used for mapping.

## 3. Results and Discussion

### 3.1. Structure and Characterization of the GO Nanoparticles

**Figure 1:**
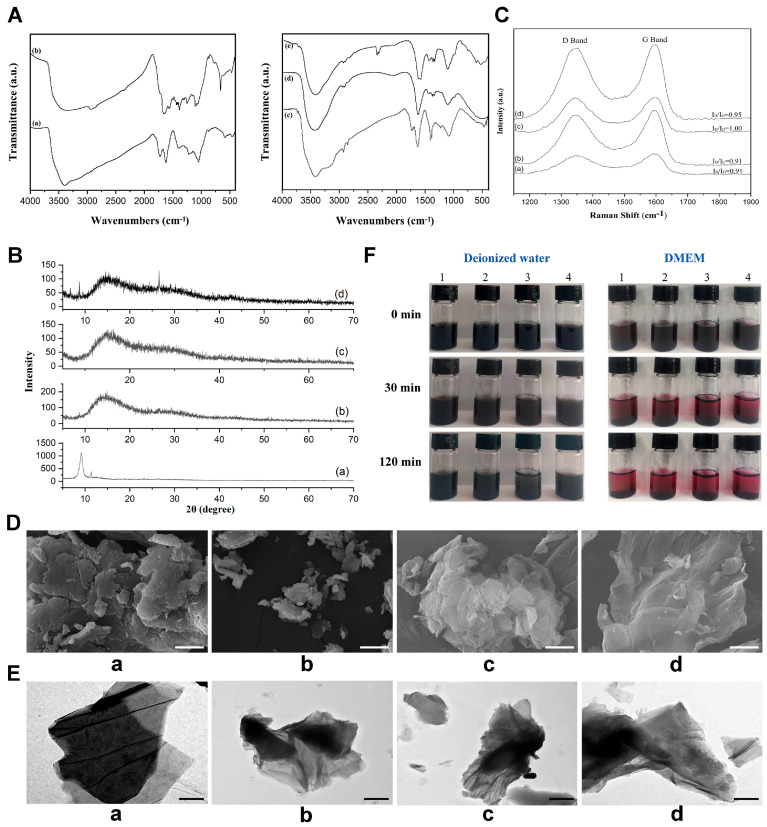
Structure and characterization of the GO and modified GOs. (**A**). FTIR spectra of GO and the modified GOs: (**a**) GO; (**b**) IPDI-GO; (**c**) PEG-GO; (**d**) PHBV-GO; (**e**) PPP-GO. (**B**). XRD patterns of GO and the modified GOs: (**a**) GO; (**b**) PEG-GO; (**c**) PHBV-GO; (**d**) PPP-GO. (**C**). Raman spectra of GO and the modified GOs: (**a**) GO; (**b**) PEG-GO; (**c**) PHBV-GO; (**d**) PPP-GO. (**D**). SEM micrographs of GO (**a**), PEG-GO (**b**), PHBV-GO (**c**) and PPP-GO (**d**), Bar = 10 μm. (**E**). TEM micrographs of GO (**a**), PEG-GO (**b**), PHBV-GO (**c**) and PPP-GO (**d**), Bar = 1 μm. (**F**). Photographs of nanocomposite suspensions in deionized water and DMEM. Images marked “0 min” represent dispersion immediately after sonication, while images marked “30 min and 120 min” are dispersion after standing still for 30 min and 120 min, respectively; 1~4 represent GO, PEG-GO, PHBV-GO and PPP-GO, respectively.

The FTIR spectra (Figure 1A) showed that GO showed its characteristic IR absorption at 3390.5 cm^−1^ (-OH), 1725.5 cm^−1^ (C = O) and 1620.0 cm^−1^ (C = C). The peaks at 1222.4 cm^−1^ and 1049.2 cm^−1^ were attributed to epoxy and alkoxy groups, respectively. IPDI-GO showed additional peaks at 2359.2 cm^−1^ of NCO and 1498.1 cm^−1^ (N–H) of NHCOO. The later peak disappeared in the modified GOs. The O–H absorption peaks of the modified GOs moved to larger wavenumbers. The asymmetric and symmetric stretching vibration of CH_3_ (2957.1 cm^−1^ and 2928.0 cm^−1^) and the stretching vibration of CH_2_ (2854.6 cm^−1^) indicated the introduction of PEG, PHBV-DIOL, and their triblock copolymer. PPP-GO showed a remarkable absorption of NCO group at 2348.2 cm^−1^ compared with the other modified GOs, perhaps owing to the large molecular weight of PPP that might relieve the reactive sites.

Fresh GO nanoparticles showed the diffraction peak (002) at 9.18° with a d-spacing value of 0.96 nm. Owing to the introduction of a large amount of hydroxyl, carboxyl, and epoxy groups, the d-spacing value increased greatly when compared with graphite (2θ = 26.5°, d-spacing value = 0.34 nm). Broad bulges centered at 2θ of 13.58° and 15.05° demonstrated the formation of amorphous phases due to the introduced PEG, PHBV, or their triblock copolymer. PPP-GO presented an additional small peak at 8.79° due to the repulsive forces that might increase the d-spacing value of GO sheets. Another peak at 26.5° illustrated the formation of the reduced GO (Figure 1B).

Raman spectroscopy (Figure 1C) is a valuable technique for analyzing the vibrational properties and microstructure of graphite crystals and different disordered graphite materials. The D and G bands are identified as the main peaks appearing at 1344 cm^−1^ and 1595 cm^−1^, respectively. These peaks are respectively related to the defect-activated breathing modes of the six membered carbon rings and the E_2g_ phonons at the Brillouin zone center, respectively. The level of disorder in the graphene substance was determined by computing the ratio of I_D_/I_G_, resulting in a value of 0.91 [26]. No distinct change in the Raman shift of the D band and G band of PEG-GO, PHBV-GO, and PPP-GO occurred. The I_D_/I_G_ ratio (0.91–1.00) is higher than that of GO (0.91), which may be due to the defects, vacancies, and deformation of the sp^2^ domain caused by the grafted polymer, resulting in the reduction of the sp^2^ domain. According to an empirical formula relating the sp^2^ cluster size (La) by Tuinstra and Koenig as Equation (1) [27], the average cluster size of GO was evaluated as 4.8 nm [28], and decreased to 4.4 nm for PPP-GO.

$$\mathrm{La}(\mathrm{nm})=\frac{10^{3}}{227\cdot \left(\frac{I_{D}}{I_{G}}\right)}$$

Under SEM, GO nanoparticles have a typical layered structure (Figure 1D(a)). The modified GOs have many wrinkles and a rough surface due to the combination of polymers, but maintain a clear layered structure (Figure 1D(b–d)). Only a few polymer clusters are present on the surface of the nanosheets. TEM results (Figure 1E) showed that GO possessed only a few layers and produced a transparent morphology. The modified GOs exhibited a darker hue compared to GO, indicating that the grafting polymer chains effectively enveloped the sharp edges of the GO sheets. GO functionalized with PEG showcased heterostructural nanohybrids resembling a sandwich formation, designed to inhibit the stacking of GO layers. Conversely, GO treated with PHBV displayed a predisposition towards aggregation. Only a few lamellars appeared at the margin of PPP-GO.

Time-dependent stability in both water and DMEM was illustrated in Figure 1F. After undergoing ultrasonic treatment, the stability of the aqueous GO suspension was maintained. This stability is attributed to the hydroxyl, carboxyl, carbonyl, and epoxide functional groups present on the basal surface or edges. These oxygen-containing groups facilitated H-bond interactions with water, maintaining stability. Notably, no aggregation was observed for GO, PEG-GO, and PPP-GO even after standing still for 120 min. However, sedimentation occurred in the aqueous PHBV-GO suspension after 30 min. Upon exposure to DMEM for 30 min, all samples precipitated, as DMEM effectively screened the electrostatic charges of the GO lamella. The characterization of GO nanomaterials also has certain limitations. We did not take into account the thickness, mass percentage, and stability time of these non-toxic polymer layers on GO, which we will investigate further to address in the future.

### 3.2. Myotoxicity Analysis of the Modified GOs Exposing to Skeletal Muscle

**Figure 2:**
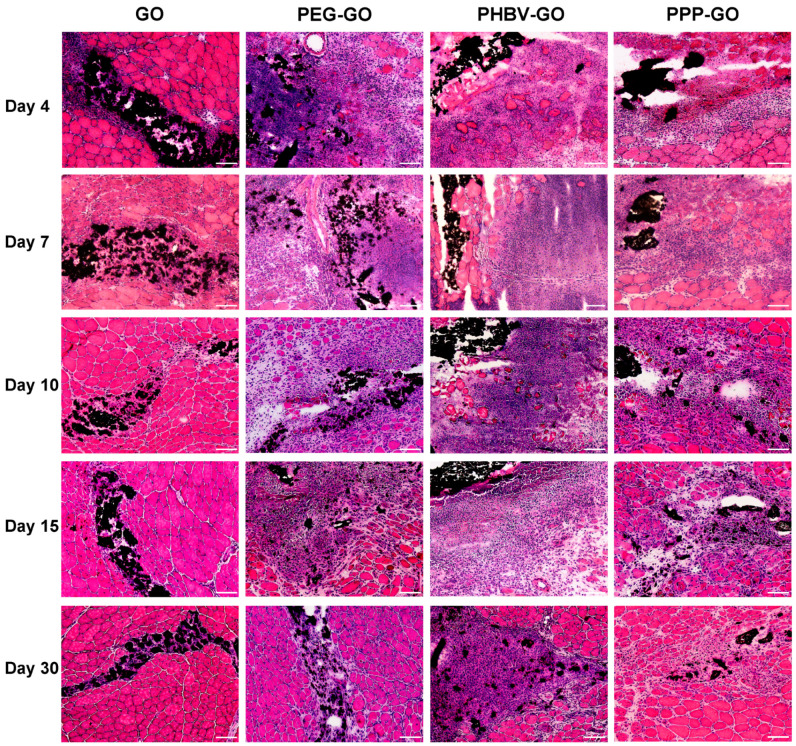
Representative H&E images of GO, PEG-GO, PHBV-GO, and PPP-GO implanted in the TA muscle of mice on days 4, 7, 10, 15, and 30. Bar = 100 μm.

Our in vitro test demonstrated that the modified GOs dispersed well in water following ultrasonic treatment. The suspensions of PEG-GO and PPP-GO showed increased stability in comparison to the PHBV-GO suspension. Sedimentation of the PHBV-GO became noticeable after 3 h of no movement (Figure 1F). PPP-GO remained stable in water due to the hydrophobic PHBV segments adhering to the GO surface and the hydrophilic PEG chains spreading out into the water [11]. Through H&E staining of the TA muscle that received the GO implants, we showed that until day 30 after implantation, the black GO nanocomposites were mainly retained within muscle (Figure 2), implying that these modified GOs should be the ideal carriers for maintaining long-term drug delivery in vivo. Notably, our observations indicate that hydrophilic PEG-GO and PPP-GO exhibited superior dispersibility compared to the hydrophobic PHBV-GO, as evidenced by the rapid infiltration of inflammatory cells and stromal fibroblasts following muscle exposure (Figure 2).

**Figure 3:**
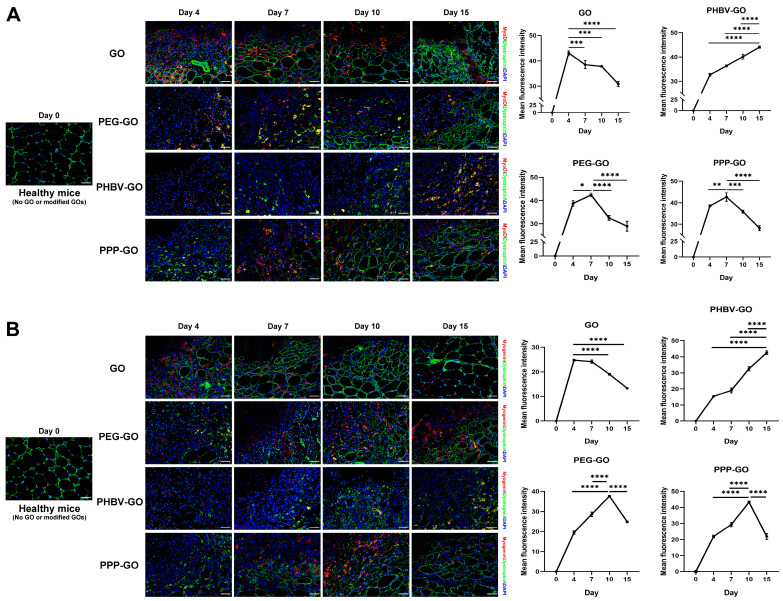
Expression of MyoD or Myogenin in regenerated muscle fibers induced by GO and modified GOs. Analysis of average fluorescence intensity of (**A**) or (**B**); One-way ANOVA was followed by Tukey’s post hoc test (* *p* < 0.05, ** *p* < 0.01, *** *p* < 0.001, **** *p* < 0.0001). All data are expressed as mean ± SD (*n* = 3 independent experiments). Bar = 50 μm.

External implantable compounds usually cause tissue necrosis and subsequent regeneration. The low toxicity of graphene-based materials in vivo has attracted more attention in biomedical applications [12,29,30]. The study focused on investigating the muscle cytotoxicity caused by the modified GO nanocarriers. It was observed that the GO were consistently present in the muscle tissue during the implantation period, leading to a slight inflammatory reaction and necrosis of muscle tissue. Significant new centronuclear myofibers with positive Dystrophin staining began to emerge on day 4, while the inflammatory response subsided by day 7. No muscle irritation could be found on day 30 post-exposure, indicating the low toxicity of the GO in vivo (Figure 2 and Figure 3). In contrast, exposure to the modified GOs caused significant intramuscular inflammatory infiltration and muscle tissue necrosis. Myofiber degeneration and inflammatory cell aggregation near PEG-GO and PHBV-GO could be observed on day 15 and day 30 post-exposure, respectively. Interestingly, PPP-GO demonstrated greater muscle compatibility compared to PEG-GO and PHBV-GO, resulting in a smaller inflammatory and necrosis zone. Inflammatory response was reduced over time, with more effective myorepair observed for PPP-GO. The necrotic zone was quickly replaced by new myofibers starting from day 15 post-exposure (Figure 2 and Figure 3). Collectively, our data indicate PPP-GO are more bio-compatible than PEG-GOs and PHBV-GO, but worse than GO in vivo, which was further verified when our in vitro culturing and differentiating myofibers received the modified GO treatment (Supplementary Figure S1). In vitro and in vivo experiments show that the good biocompatibility of PPP-GO may provide new ideas for researching new drug delivery systems.

In the co-culture system of ATDC5 cells and GO, we observed that fewer dead cells appeared in the modified GOs than in GO, indicating that polymer modification can reduce the cytotoxicity of GO [11]. In vitro studies have shown that GO-based compounds can be used as suitable scaffolds to promote the attachment, proliferation, and differentiation of skeletal muscle precursor cells [31,32,33]. However, controversial results were obtained regarding the toxicity of GO and the modified GOs in muscle. We speculate that, after muscle exposure, the coating of biocompatible polymers on GO is more likely to provoke muscle necrosis and local inflammation infiltration, implying the toxicity of the modified GOs in vivo. This requires further study to better understand how myotoxicity occurs.

### 3.3. Myogenesis Comparison of Muscle Exposing to the Modified GOs

After implantation of GO nanomaterials, we observed using histological staining that the muscle necrosis induced by the modified GO was replaced by slow muscle fiber regeneration (Figure 2 and Figure 3). By employing fluorescent double-staining of Dystrophin and either MyoD, Myogenin, Desmin, or embryonic myosin heavy chain (eMyHC)—all crucial early indicators of myogenesis—we conducted a detailed comparison of the myofiber regeneration in muscles treated with the modified GOs (Figure 3, Supplementary Figure S2).

We have noticed the GO had low toxicity in vivo, and this has now been further supported, since at the early stages of the implantation (day 4), we observed a remarkable aggregation of new myofibers in GO-exposed muscle, which presented a clear expression of Dystrophin and MyoD, Myogenin, eMyHC, or Desmin (Figure 3, Supplementary Figure S2). Otherwise, lesser new myofibers appeared in the modified GO-treated muscle, with the postponed protein expression of the above molecules in myofiber. In truth, muscle samples exposed to PEG-GO and PPP-GO showed a significant emergence of new, centrally nucleated myofibers (Dystrophin^+^) starting from day 7, at which point the MyoD protein expression peaks. The protein expression peak of Myogenin, eMyHC, and Desmin appeared on day 10 post-implantation. However, the expression peak of eMyHC, Desmin, MyoD and Myogenin in muscles exposed to PHBV-GO was delayed until day 15 post-implantation (Figure 3, Supplementary Figure S2). Markers of early myogenesis, including MyoD, Myogenin, Desmin, and eMyHC exhibit high expression levels in newborn muscle fibers. As muscle fibers mature during the process of muscle repair, their expression is decreased [34,35]. Our data thus suggest that muscle regeneration in hydrophilic PEG-GO and PPP-GO-treated muscle was earlier and more effective than in hydrophobic PHBV-GO-treated muscle. The benefits of GO-based platforms for skeletal muscle regeneration have been validated through the direct differentiation of C2C12 myoblasts [33], and the transformation of human cord blood-derived pluripotent mesenchymal stem cells (CB-hMSCs) into myofibers in vitro [32]. Our data evidenced that GO primed the formation of multinucleated myotubes after muscle exposure. Furthermore, we proved the better bio-compatibility of PPP-GO than PEG-GO in vivo, since the above proteins (eMyHC, MyoD, Myogenin) were expressed at lower levels than PEG-GO in muscles exposed to PPP-GO on day 15 (Figure 3, Supplementary Figure S2).

### 3.4. Comparison of Intramuscular Inflammation Induced by the Modified GOs

**Figure 4:**
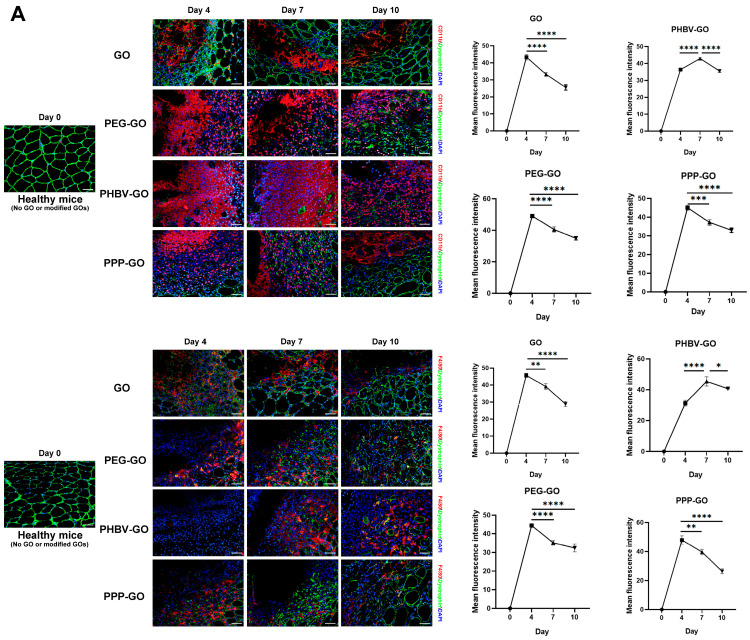

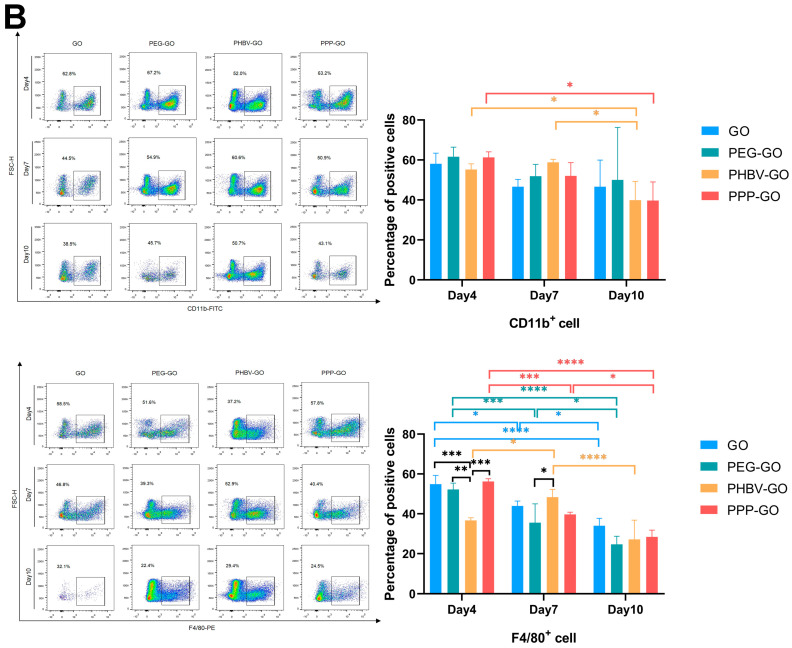

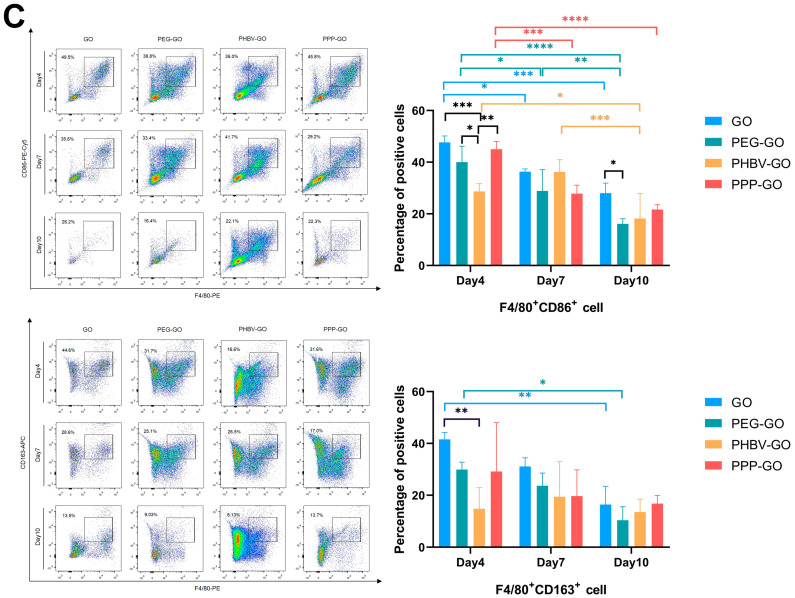
GO and modified GOs induce mononuclear/macrophage infiltration into transplanted muscles. (**A**). Immunofluorescence staining of TA muscle for CD11b or F4/80, Bar = 50 μm. Analysis of average fluorescence intensity of CD11b or F4/80; One-way ANOVA was followed by Tukey’s post hoc test (* *p* < 0.05, ** *p* < 0.01, *** *p* < 0.001, **** *p* < 0.0001); (**B**,**C**). FACS analysis of mononuclear/macrophage and M1/M2 macrophage ratios in TA muscle after GO and modified GOs implantation. Two-way ANOVA was followed by Tukey’s post hoc test (* *p* < 0.05, ** *p* < 0.01, *** *p* < 0.001, **** *p* < 0.0001). All data are expressed as mean ± SD (*n* = 3 independent experiments).

In muscle after specific stimulation or injury, monocytes and macrophages dominate the basic inflammatory response [29,36]. Therefore, we next performed immunofluorescence staining to assess the infiltration of these cells after the GO material was implanted into the muscle. We defined monocyte as CD11b^+^ cells and macrophages as F4/80^+^ cells. As shown in Figure 4A, macrophage withdrawal was quicker in GO-treated muscle, and on day 10 post-implantion, CD11b^+^ or F4/80^+^ cells could barely be found near the GO. Of note, when we turned to the modified GOs, we found that in PHBV-GO, the peak of monocyte/macrophage invasion occurred at about day 7. For PPP-GO and PEG-GO, the peak of CD11b^+^ or F4/80^+^ cell infiltration was observed on day 4 after implantation and decreased rapidly on day 7.

Through FACS analysis, we further demonstrated that the material exposure induced a notable intramuscular infiltration of CD11b^+^ or F4/80^+^ cells. Compared with GO, PEG-GO, and PPP-GO, PHBV-GO induced less mononuclear/macrophage accumulation during early implantation (day 4). On day 7, macrophages peaked in PHBV-GO-treated muscle, but rapidly decreased in GO-, PEG-GO- and PPP-GO-treated muscle (Figure 4B). GOs tended to aggregate together and only expose a small amount of polymer clusters on the surface. PPP-GO decreased the spreading area by turning into the micelle [11]. PEG-GO has excellent hydrophilic properties. We presumed that these features were attributed to the transient muscle macrophage invasion by GO, PEG-GO and PPP-GO. Interestingly, when we turned to compare the percentage of the pro-inflammatory M1 (F4/80^+^CD86^+^) and the pro-resolving M2 macrophage (F4/80^+^, CD163^+^) in muscle involved in the implant, we found that on day 4, GO, PEG-GO, and PPP-GO induced significant M1 macrophage aggregation, followed by a gradual decline (Figure 4C). Conversely, PHBV-GO induced less M1 macrophage aggregation in the early stage (Figure 4C). Han et al. reported that GO can be used as a natural antioxidant to reduce the inflammatory polarization of M1 macrophages by reducing ROS in macrophages, and can also be used as a gene carrier to further polarize M1 macrophages into anti-inflammatory M2 macrophages, so as to synergically treat myocardial infarction [37]. Our findings indicated that the addition of PEG or PHBV DIOL to the surface of GOs, or the enhancement of graphene layers in the modified GOs, had no significant impact on M1 or M2 macrophages.

**Figure 5:**
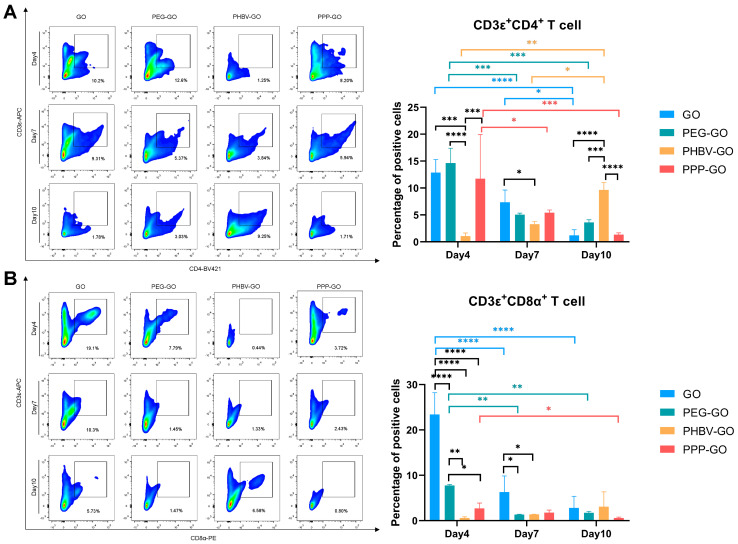
FACS analysis of T cell infiltration induced by GO and modified GOs into transplanted muscles. (**A**,**B**). The proportion of CD4^+^, CD8^+^ T cells; Two-way ANOVA was followed by Tukey’s post hoc test (* *p* < 0.05, ** *p* < 0.01, *** *p* < 0.001, **** *p* < 0.0001). All data are expressed as mean ± SD (*n* = 3 independent experiments).

Modified GOs may degrade or release special components that act as foreign immunogens to trigger secondary immunity in vivo. Recently, Parker et al. reported that in vitro, small graphene oxide (sGO) sheets can enhance the ability of DCs to process and/or present the protein Ag, thereby activating CD4^+^ T cells [38]. Through intravenous injection of graphene oxide, Wu HY et al. found that PEG-GO could significantly reduce the production of antigen-specific IgE and enhance the metabolic activity of spleen cells and the expression of Th1 and Th2 cytokines [39]. In order to further investigate the antigenicity of GO and modified-GOs, we used FACS analysis to monitor the infiltration of T cells at the muscle implant site. As shown in Figure 5A, the GO and the modified GOs attracted CD4^+^ T cells to invade the muscle on day 4 post-implantation. On day 7, we found rapid withdrawal of CD4^+^ T cells from GO, PEG-GO, and PPP-GO coated muscles, but slower withdrawal from PHBV-coated muscles. On day 10, the infiltration of CD4^+^ T cells in PHBV-GO-coated muscles reached a peak. Unexpectedly, we did not observe a significant difference in CD4^+^ T cell percentage between PEG-GO and PPP-GO when comparing the three modified GOs. This suggests that the degraded components of modified GOs, especially those of PEG or PPP, have the same chemotactic capacity.

In vitro, GO flakes can improve the capacity of dendritic cells to process and/or present proteins or peptide antigens, thereby activating CD8^+^ T cells towards a cytotoxic T lymphocyte phenotype [38]. We next turned to CD8^+^ T cell infiltration in the nanocomposite exposed muscle, and we found that CD8^+^ T cells had slight infiltration in modified GO exposed muscle compared to GO. We monitored the faster withdraw of CD8^+^ T cells in GO and PEG-GO exposed muscle (Figure 5B). However, during the progression of the inflammation, CD8^+^ T cell infiltration induced by PPP-GO implantation was minimal, while the number of CD8^+^ T cells induced by PHBV-GO tended to increase. Overall, our work thus indicates that the modified GOs are relatively bio-compatible and only induced a weak cytotoxic T cell response. In addition, we have monitored that PEG-GO is more sensitive to CD8^+^ T cells than other modified GOs (Figure 5B), and special attention should be paid to this when further modifying GO in the future in order to more effectively reduce biological reactivity.

### 3.5. Aging Related Features of PPP-GO Induced Myo-Reactivity

In recent years, with the progress of the economy and medical care, the proportion of elderly people in the population continues to grow. Aging is accompanied by a variety of physiological processes, such as decreased immunity, slower basic metabolism, and decreased activity of antioxidant-related enzymes [40]. Therefore, elderly people are usually requiring more medication interventions. At present, a GO carrier targeted-drug delivery system has been proposed as an effective and safe therapy for age-related disorders, such as Parkinson’s disease [41] and ischemic heart disease [42]. Thus, there is a desperate need to consider the in vivo reaction characteristics of chemically modified GOs for the elderly. We have previously demonstrated that PPP-GO have similar dispersion and stability to PEG-GO and higher hydrophobic drug carrying capacity than PHBV-GO, indicating its superior properties to homopolymers [11]. So far in this work, through in vitro and in vivo experiments, we had demonstrated that PPP-GOs are more bio-compatible than PEG-GO and PHBV-GO when exposed to muscle. Based on these results, further studies on the biosafety of PPP-GO in older adults are needed. We focused on the issue and addressed the muscle response difference induced by PPP-GO exposure to the TA muscle in adult and old mice.

As presented in Figure 2 and Supplementary Figure S3A, we noticed over the period of implantion, PPP-GOs dispersed more quickly in the muscle of young adult mice than in old mice. On day 15, the aggregation area of PPP-GO within young muscle was only about half of the aged muscle. Our data thus suggest the decreased skeletal muscle cleaning ability of the exogenous PPP-GO in elderly animals. Interestingly, in young adult mice, PPP-GO induced myocyte degeneration was rapidly replaced by the new centronuclear myofibers(dystrophin^+^). On day 15, PPP-GOs were almost completely surrounded by new myofibers. On the contrary, in aged mice, myofiber regeneration was delayed, demonstrated by the lesser number and smaller diameter of new myofibers compared to young mice (Figure 3, Supplementary Figures S2 and S3B). Dystrophin and MyoD, Myogenin, eMyHC, or Desmin were detected by fluorescence double staining. We further verified that aged mice require a longer time to complete myorepair of the necrotic muscle provoked by the implanted PPP-GO (Figure 3, Supplementary Figures S2 and S3B,D). In young adult mice, we detected a notable inflammation response provoked by the PPP-GO, but inflammation response resolved rapidly. Notably, when we turned to old mice, we found that the inflammatory response caused by PPP-GO exposure in the muscles of old mice was relatively mild on day 4 compared to young mice, but the expression of F4/80 or CD11b was higher than in young mice on day 7 or 10 after treatment. We suggest that inflammatory cells in old mice have a delayed response to implant stimulation (Figure 4, Supplementary Figure S3C,D).

**Figure 6:**
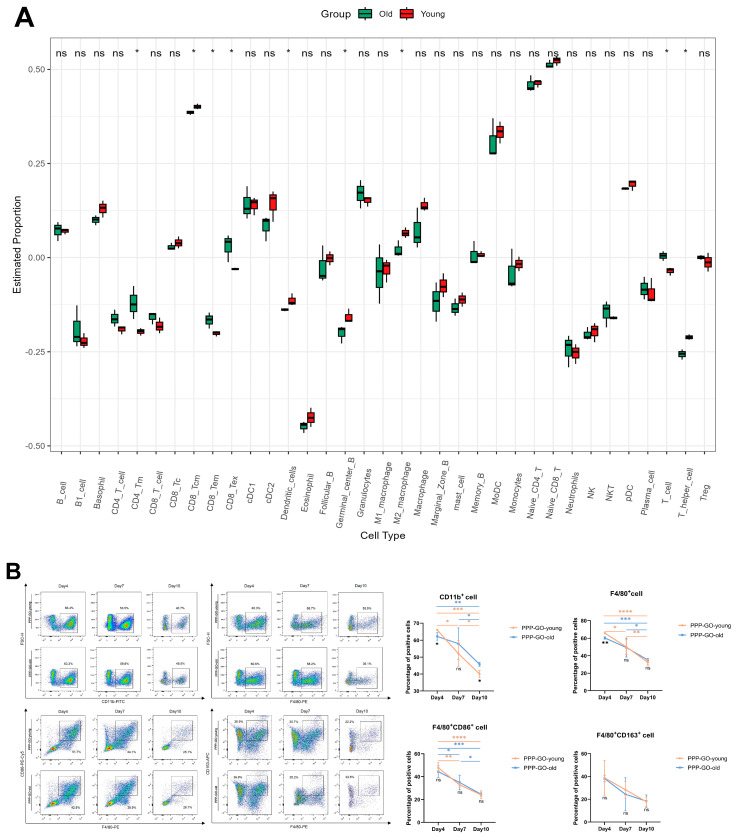

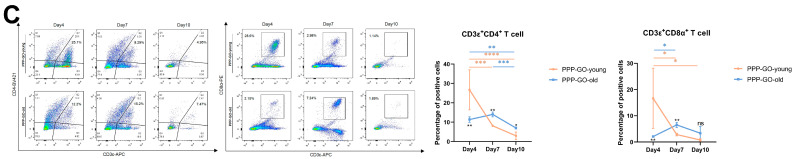
Comparison of immune cell infiltration in old mice and young mice after PPP-GO muscle transplantation. (**A**). ssGSEA of young and old mice on day 4 after BaCl_2_ injury. (**B**). FACS analysis of PPP-GO induced infiltration of mononuclear/macrophage and M1/M2 macrophage in transplanted young or old mice muscles. (**C**). FACS analysis of PPP-GO induced infiltration of CD4^+^ or CD8^+^T cells in transplanted young or old mice muscles. Two-way ANOVA was followed by Sidak’s post hoc test (ns: *p* > 0.05, * *p* < 0.05, ** *p* < 0.01, *** *p* < 0.001, **** *p* < 0.0001). All data are presented as mean ± SD (*n* = 3 independent experiments).

In order to further analyze the immune cell infiltration of young or old mice implanted with PPP-GO, David E. Lee’s analysis of immune infiltration of young and old mice on day 4 of muscle injury by BaCl_2_ provided us with directions [43]. Therefore, we performed a ssGSEA (the source data is GSE217037). According to ssGSEA (Figure 6A), we found significant differences in T cells and M2 macrophages of injured muscle between old and young mice (Figure 6A). Therefore we selected these two types of cells for further analysis by FACS. In both young and old mice, mononuclear/macrophage infiltration peaked on day 4, followed by a rapid decrease in the proportion of CD11b^+^ or F4/80^+^ cells, but there was no significant difference in the proportion of M1 or M2 macrophages between young and old mice (Figure 6B). Surprisingly, the young mice induced significant CD4^+^ and CD8^+^ T cell infiltration on day 4 of PPP-GO implantation, which was significantly higher than that of the older mice, followed by a rapid decline. The infiltration of T cells in old mice reached a peak on day 7, and the proportion was significantly higher than that of young mice, indicating that the induction of CD4^+^ and CD8^+^ T cell infiltration in old mice was delayed. (Figure 6C). Collectively, our data suggested that aged animals are more vulnerable to exogenous stress, illustrated by a delayed inflammatory response and muscle regeneration, even when receiving compatible GO material in muscle. However, in both old and adult mice, GO nanoparticles may also trigger specific reactions in different organs, including the heart, liver, lungs, kidneys, and so on. We will further evaluate the effects of nanoparticles on other organs in future work.

## 4. Conclusions

The GO was chemically modified with PEG, PHBV, or PPP. The stability of the PPP-GO and PEG-GO suspensions after ultrasonic treatment is higher compared to that of PHBV-GO. Implantation of modified GO into the muscle induces varying degrees of intramural inflammation and subsequent myorepair, with PPP-GO demonstrating superior biocompatibility over PEG-GO and PHBV-GO. Therefore, PPP-GO holds potential as a high-quality drug delivery system in future applications. When implanted into the muscles of old or young animals respectively, PPP-GO exhibited more pronounced effects on older animals.

## Data Availability

The original contributions presented in the study are included in the article/Supplementary Material, further inquiries can be directed to the corresponding authors.

## Supplementary Materials

The following supporting information can be downloaded at: https://www.mdpi.com/article/10.3390/jfb16040115/s1, Figure S1: Representative white light image of GO and modified -GOs cocultured with C2C12 differentiated myotubes for 24 h; Figure S2: Expression of eMyHC or Desmin in regenerated muscle fibers induced by GO and modified GOs; Figure S3: H&E and immunofluorescence images of PPP-GO implanted into TA muscles of old mice and compared with young mice.
