# Supplementary material for: Intramuscular Reactivity of the Modified Graphene Oxides and Their Bio-Reactivity in Aging Muscle
Source: J Funct Biomater. 2025 Mar 25;16(4):115. doi: 10.3390/jfb16040115 (PMC12027639; doi:10.3390/jfb16040115)
Supplement: Supplementary file 1 [file jfb-16-00115-s001.zip › jfb-3451788-supplementary.pdf]

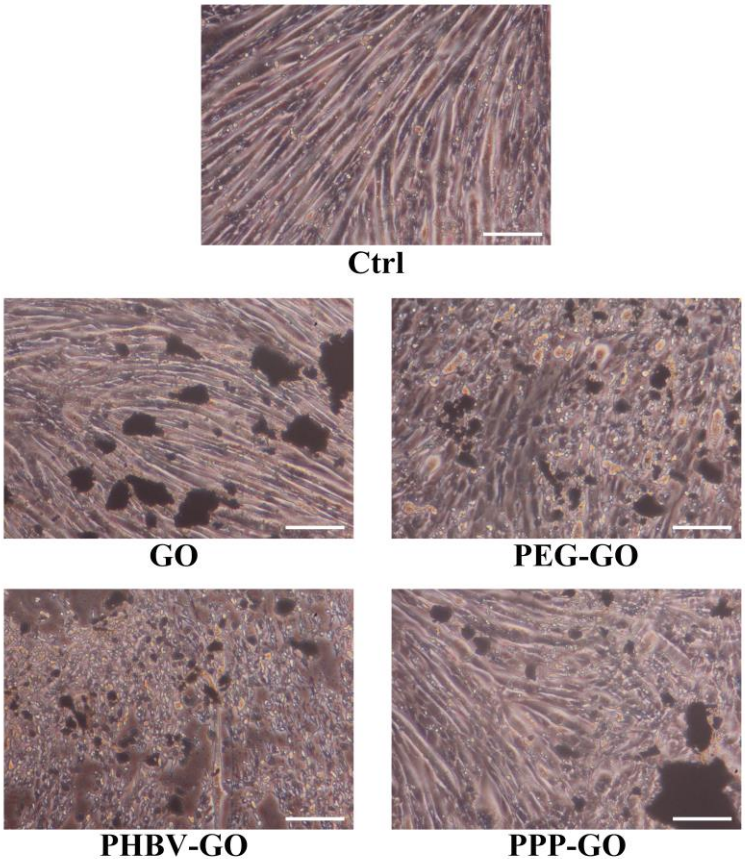

Supplementary Figure S1. Representative white light image of GO and modified -GOs cocultured with C2C12 differentiated myotubes for 24h. Bar=200 μm.

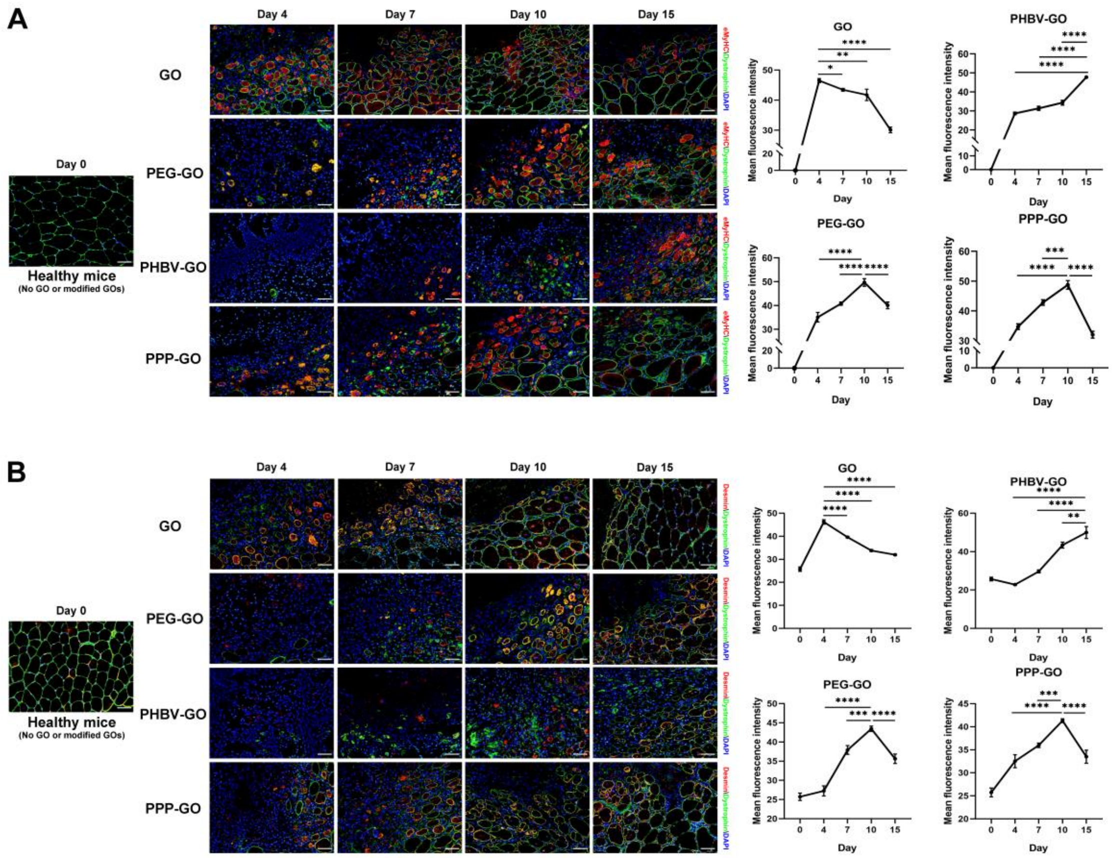

Supplementary Figure S2. Expression of eMyHC or Desmin in regenerated muscle fibers induced by GO and modified GOs. Analysis of average fluorescence intensity of (A) or (B); One-way ANOVA was followed by Tukey's post hoc test (* $p < 0.05$, ** $p < 0.01$, *** $p < 0.001$, **** $p < 0.0001$). All data are expressed as mean ± SD ($n = 3$ independent experiments). Bar = 50 μm.

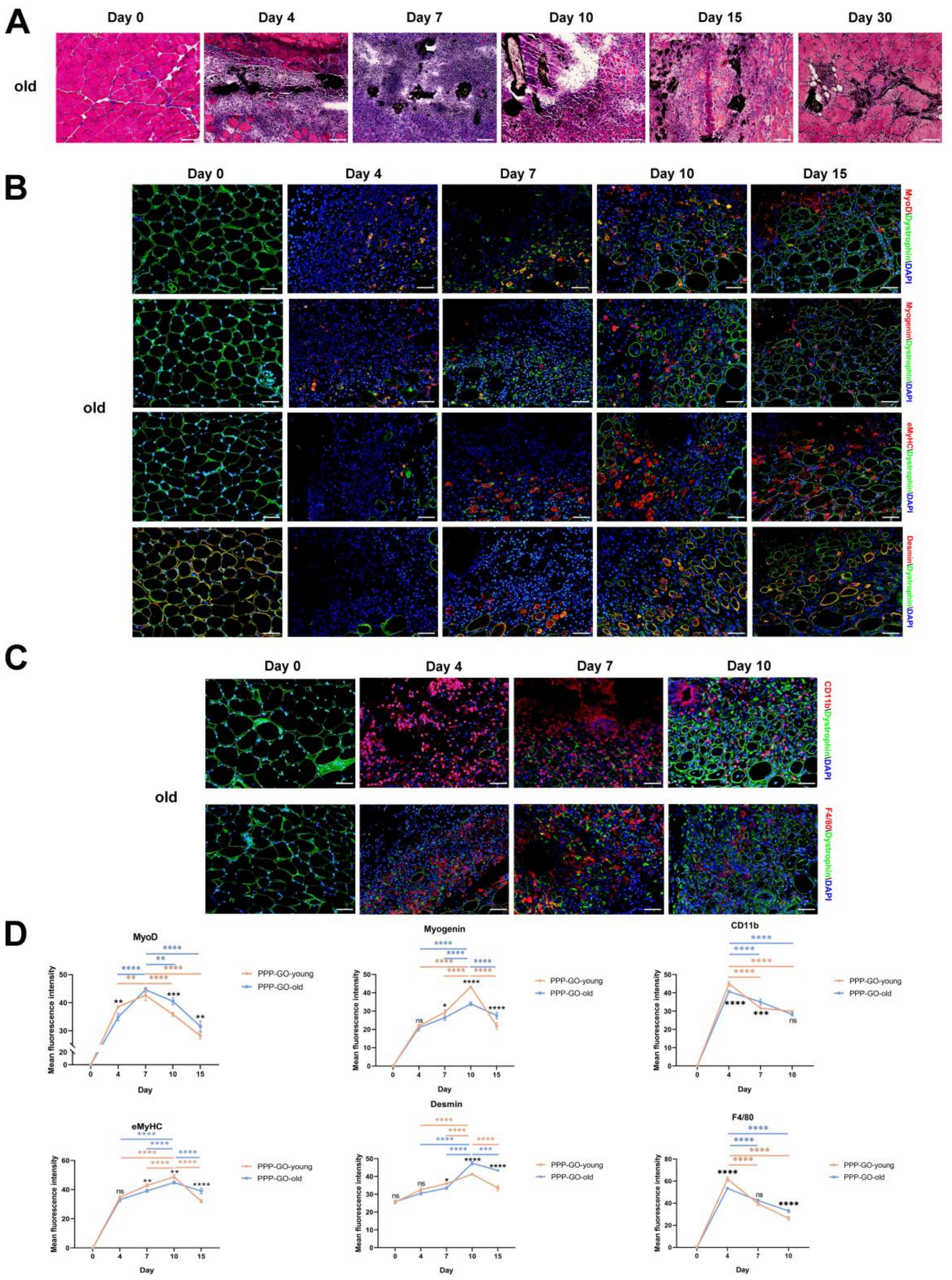

Supplementary Figure S3. H&E and immunofluorescence images of PPP-GO implanted into TA muscles of old mice and compared with young mice. (**A**). Representative H&E images of TA muscles in old mice were implanted with PPP-GO. Bar = 100μm. (**B**,**C**). Expression of MyoD, Myogenin, eMyHC, Desmin, CD11b or F4/80 in TA muscle of PPP-GO transplanted into old mice; Bar = 50μm. (**D**). Comparison of mean fluorescence intensity of MyoD, Myogenin, eMyHC, Desmin, CD11b or F4/80 in TA muscle of young and old mice implanted with PPP-GO; Two-way ANOVA was followed by Sidak's post hoc test (ns: $p > 0.05$, $*p < 0.05$, $**p <$

0.01, ***$p < 0.001$, ****$p < 0.0001$). All data are presented as mean ± SD ($n = 3$ independent experiments).
